# Tetrandrine inhibits RANKL-induced osteoclastogenesis by promoting the degradation of TRAIL

**DOI:** 10.1186/s10020-022-00568-4

**Published:** 2022-11-26

**Authors:** Jiarui Li, Xiang Li, Shengji Zhou, Yuxin Wang, Yang Lu, Quan Wang, Fengchao Zhao

**Affiliations:** Department of Orthopaedic Surgery, The First Affiliated Hospital, Zhejiang University School of Medicine, No.79 Qingchun Road, Hangzhou, 310003 People’s Republic of China

**Keywords:** Tetrandrine, TRAIL, RANKL, Osteoclastogenesis, OVX

## Abstract

**Background:**

Tetrandrine, a bisbenzylisoquinoline (BBI) alkaloid extracted from *Stephania tetrandra* (S. Moore), and is widely used in several diseases such as tuberculosis, hyperglycemia, malaria, and tumors. Tetrandrine was recently shown to prevent bone loss in ovariectomized mice. However, the specific mechanism underlying osteoclastogenesis inhibition remains unclear.

**Methods:**

Tetrandrine’s cytotoxicity to cells was determined using the Cell Counting Kit-8 assay. Tartrate-resistant acid phosphatase staining, immunofluorescence and bone resorption assay were performed to evaluate osteoclasts’ differentiation and absorption capacity. The bone-forming capacity was assessed using alkaline phosphatase and Alizarin red S staining. qPCR and Western blotting were applied to assess the related genes and protein expression. Tetrandrine’s impact on TRAIL was demonstrated through a co-immunoprecipitation assay. Animal experiments were performed for the detection of the therapeutic effect of Tetrandrine on osteoporosis.

**Results:**

Tetrandrine attenuated RANKL-induced osteoclastogenesis and decreased the related gene expression. The co-immunoprecipitation assay revealed that Tetrandrine administration accelerated the ubiquitination of TNF-related apoptosis-inducing ligand (TRAIL), which was subsequently degraded. Moreover, TRAIL overexpression was found to partially reverse the Tetrandrine-induced inhibition of osteoclastogenesis. Meanwhile, Tetrandrine significantly inhibited the phosphorylation of p38, p65, JNK, IKBα and IKKα/β, while the TRAIL overexpression weakened this effect. In addition, Tetrandrine promoted osteogenesis and inhibited the TRAIL expression in osteoblasts. Tetrandrine consistently improved bone destruction by stimulating bone formation and inhibiting bone resorption in an OVX-induced mouse model.

**Conclusion:**

Tetrandrine inhibits RANKL-induced osteoclastogenesis by promoting TRAIL degradation and promotes osteoblast differentiation, suggesting its potential in antiosteopenia pharmacotherapy.

## Background

The human skeleton is constantly being rebuilt and it is precisely regulated by osteoclasts, osteoblasts and osteocytes (Raggatt and Partridge 2010). Osteoclasts are myeloid lineage multinucleated giant cells that absorb bone, while osteogenesis regulates calcium salt deposition. Osteoporosis is caused by both the abnormal activation of osteoclasts and the dysfunction of osteoblasts (Fujii et al. 2021; Guo et al. 2019). According to statistics, about 19% of men and 30% of women in Europe and the United States are at risk of osteoporosis every year, respectively, with over 9 million osteoporosis-related fractures occurring every year (Migliorini et al. 2021). Postmenopausal osteoporosis is the most common cause of osteoporosis. The major cause of postmenopausal osteoporosis is the reduction in estrogen production in the postmenopausal ovary. When the estrogen levels are reduced, osteoclast differentiation and activity increases, whereas the osteoblast differentiation and activity decreases (Eastell et al. 2016). Postmenopausal osteoporosis is becoming prevalent as the global population ages. It is expected to seriously affect the quality of life of elderly women and increase the burden on society.

Mononuclear macrophages derived from bone marrow progenitor cells in bone marrow usually merge into osteoclasts, which are the only cells in the human body that play a role in bone resorption when co-stimulated with macrophage colony-stimulating factor (MCSF) and receptor activator of nuclear factor-κB ligand (RANKL) (Chen et al. 2019; Li et al. 2021). The primary strategy for preventing and inhibiting osteoporosis is to reduce or control bone resorption. The osteoprotegerin (OPG)/receptor activator of the NF-κB (RANK)/RANKL signaling pathway is essential for osteoclastogenesis regulation. RANKL interacts with its receptor RANK to recruit TNF receptor related factor 6 (TRAF6), which subsequently activates the downstream effectors such as nuclear factor-kB (NF-κB) and mitogen activated protein kinases (MAPKs), leading to the activation of nuclear factor of activated T cells 1 (NFATc1) (Yamashita et al. 2007). NFATc1 is a terminal regulator of osteoclast differentiation that affects osteoclast differentiation and the expression of osteoclast-related genes, such as tartrate-resistant acid–base phosphatase (TRAP), cathepsin K (CTSK), and dendritic cell-specific transmembrane protein (DC-STAMP) (Boyce 2013; Negishi-Koga and Takayanagi 2009; Takayanagi 2007). However, OPG, as the decoy receptor of RANKL, inhibits osteoclasts activation (Eastell et al. 2016; Jiang et al. 2021; Trouvin and Goëb 2010). Denosumab is a RANKL inhibitor that is used in the treatment of osteoporosis. While it is the strongest drug to inhibit bone resorption, long-term usage will cause rash, infection and atypical femoral fracture (Palmerini et al. 2017). Therefore, finding a drug that can effectively treat osteoporosis while having fewer adverse effects is critical.

Tetrandrine is a bisbenzylisoquinoline (BBI) alkaloid extracted from *Stephania tetrandra* (S. Moore) that is widely applied in several diseases including tuberculosis, hyperglycemia, malaria, and tumor (Liu et al. 2016; Bhagya and Chandrashekar 2018). Xu et al. (2021) discovered that Tetrandrine inhibits the growth of activated T cells via a variety of signaling pathways including NF-κB, caspase cascade, cell cycle, MAPK, and PI3K/Akt/mTOR, to inhibit the growth of T-cell acute lymphoblastic leukemia. In addition, Gao et al. (2016) confirmed that Tetrandrine, as an anti-inflammatory drug, can inhibit the NF-κB pathway by inhibiting IκBα and NF-κB p65 phosphorylation, thereby reducing the release of proinflammatory cytokines. Furthermore, in terms of osteogenesis, Tetrandrine is considered to promote the proliferation of osteoblasts by blocking a large conductivity (BK) potassium channel (Henney et al. 2009). Tetrandrine has recently been proven in experiments to prevent bone loss in ovariectomized mice (Shi et al. 2022; Zhong et al. 2019). However, to further explore the specific mechanism of Tetrandrine in inhibiting osteoporosis, a further in-depth research is warranted.

TNF-related apoptosis-inducing ligand (TRAIL) is a member of the tumor necrosis factor (TNF) superfamily. As per past reports, it can induce apoptosis in a variety of tumor cells, including osteosarcoma cells, without affecting most human cell types (Gamie et al. 2017; Pan et al. 2013). The research by Yen et al. proved that TRAIL triggered osteoclast differentiation via a (necrosis factor receptor-associated factor-6) TRAF-6 dependent signal pathway, as also confirmed (Gao et al. 2021; Yen et al. 2012). Other experiments confirmed that microgravity induced the TRAIL expression in preosteoclast cells and promoted osteoclast differentiation (Sambandam et al. 2016). In addition, TRAIL can suppress OPG-mediated osteoclast formation (Emery et al. 1998).

Through our experiment, we aimed to explore the role of Tetrandrine in osteoclast and osteoblast formation, as well as their relationship with the TRAIL-TRAF6-NF-κB/MAPKs signal axis, with the hope to determine a valuable method for preventing and treating osteoclast-related diseases.

## Methods

### Cell isolation and culture

C57BL/6 mice aged 8 weeks were sacrificed by cervical dislocation to extract bone marrow macrophages (BMMs). The mouse corpse was disinfected with 75% alcohol, and the femur and tibia were separated, along with the surface muscle tissue. The bone marrow was then flushed into a 10-cm dish. At 37 °C and under 5% CO2 incubation, the cells were cultivated in an α-MEM medium (Gibco-BRL, Gaithersburg, MD, USA) supplemented with 10% FBS (Gibco-BRL), 1% penicillin/streptomycin (1%, Gibco-BRL), and 30 ng/mL M-CSF (R&D, Minneapolis, MN, USA). After 48 h, the medium was changed to eliminate the unattached cells, and the attached cells were used for subsequent experiments. Mouse embryonic fibroblasts (C3H10) were purchased from ATCC (Manassas, VA, USA) and cultured in an incubator (37 °C, 5% CO_2_). The growth medium was DMEM supplemented with 10% FBS and 1% penicillin/streptomycin.

### Cell Counting Kit-8 assay

The CCK-8 test was used to evaluate Tetrandrine’s cytotoxicity on BMMs and C3H10. Briefly, 1 × 10^5^ cells were added to each well of a 96-well plate and cultivated for 48 and 96 h with Tetrandrine (Selleck, Houston, Texas, United States). The CCK8 reagent (10 uL) (Dojindo Molecular Technology, Kumamoto, Japan) was then added to each well and cultured for an hour. The absorbance at 450 nm was measured by a microplate reader (Bio-Tek Instruments, Winooski, VT, USA).

### In vitro osteoclastogenesis assay

BMMs (5 × 10^3^) were added to each well of a 96-well plate and incubated overnight. BMMs were then incubated with 30 ng/mL M-CSF and 50 ng/mL RANKL (R&D), with or without Tetrandrine at a certain concentration (0, 0.25, 0.5, and 1 μM). The medium was changed every 2 days for 7 days, and the cells were then rinsed twice with PBS before fixing with 4% paraformaldehyde (PFA) at room temperature for 15 min, and finally stained with tartrate-resistant acid phosphatase (TRAP) (Sigma-Aldrich, St. Louis, MO, USA) for 30 min.

### Bone resorption assay

The bottom of a 96-well plate was filled with bovine bone slices (Corning Inc., Corning, NY, USA), and 1 × 10^4^ BMMs were seeded in each well. After 24 h, BMMs were differentiated with 30 ng/mL of M-CSF, 50 ng/mL RANKL, and different concentrations of Tetrandrine (0, 0.5, and 1 μM). The medium was changed every 2 days until the 7th day. After osteoclast differentiation, the bovine bone slices were removed and rinsed thrice with PBS. After drying the bone slices, we photographed them with a TM1000 desktop scanning electron microscope (Hitachi High-Technologies, Tokyo, Japan) and the bone absorption area was analyzed with ImageJ software (NIH, Bethesda, MD, USA).

### Osteoblast differentiation

C3H10 cells were osteogenically induced in a DMEM medium comprising 50 μg/mL ascorbic acid and 10 μM β-glycerophosphate (Sigma-Aldrich). After osteogenic induction for 7 days, alkaline phosphatase (ALP) staining was performed with BCIP/NBT kit (CWBIO, Beijing, China), and the ALP activity was quantified by an alkaline phosphatase detection kit (Beyotime, Nanjing, China). After 14 days of osteogenic induction, alizarin red S (ARS) staining was performed with 1% alizarin red staining solution (Sigma-Aldrich) to evaluate the calcium deposition in the extracellular matrix. To quantify the mineralization, staining was eluted with 10% glacial acetic acid and the absorbance was measured at 405 nm by a microplate reader (Bio-Tek Instruments).

### Cell transfection

BMMs were harvested into 6-well or 12-well plates, and the medium was replaced every 2 days. When the cell density reached 50–60%, they were stimulated with RANKL for 4 h before being transfected with TRAIL siRNA, TRAIL vector, or negative controls (control siRNA and control vector) using the TSnanofect transfection reagent (Tsingke Biotechnology Co, Beijing, China) according to the manufacturer’s instructions. Tsingke Biotechnology Co. supplied TRAIL siRNA, TRAIL vector, and negative controls (control siRNA and control vector). The TRAIL siRNA sequences are listed in Table 1.

**Table 1:** Sequences of primers and siRNA

Primer	5′-3′
GAPDH-F	ACCCAGAAGACTGTGGATGG
GAPDH-R	CACATTGGGGGTAGGAACAC
CTSK-F	CTTCCAATACGTGCAGCAGA
CTSK-R	TCTTCAGGGCTTTCTCGTTC
c-Fos-F	CCAGTCAAGAGCATCAGCAA
c-Fos-R	AAGTAGTGCAGCCCGGAGTA
NFATc1-F	CCGTTGCTTCCAAAAATAACA
NFATc1-R	TGTGGGATGTGAACTCGGAA
DC-STAMP-F	AAAACCCTTGGGCTGTTCTT
DC-STAMP-R	AATCATGGACGACTCCTTGG
TRAP-F	TCCGTGCTCGGCGATGGACCAGA
TRAP-R	CTGGAGTGCACGATGCCAGCGACA
TRAIL-F	ACCTCAGAAAGTGGCAGCTC
TRAIL-R	CTGCACCAGCTGTTTGGTTC
Runx2-F	TTCTCCAACCCACGAATGCAC
Runx2-R	CAGGTACGTGTGGTAGTGAGT
OPG-F	TGGAGATCGAATTCTGCTTG
OPG-R	TCAAGTGCTTGAGGGCATAC
OCN-F	GAGGGCAATAAGGTAGTGAACAGA
OCN-R	AAGCCATACTGGTTTGATAGCTCG
ALP-F	CCAACTCTTTTGTGCCAGAGA
ALP-R	GGCTACATTGGTGTTGAGCTTTT

siTRAIL#1-F	GUCAGCACUUCAGGAUGAUTT
siTRAIL#1-R	AUCAUCCUGAAGUGCUGACTT
siTRAIL#2-F	CAGUACAUCUACAAGUACATT
siTRAIL#2-F	UGUACUUGUAGAUGUACUGTT
siTRAIL#3-F	GGUCCAGAGAUGCCGAGUATT
siTRAIL#3-F	UACUCGGCAUCUCUGGACCTT

### RNA extraction and quantitative real-time polymerase chain reaction (qPCR)

TRIzol Reagent (CWBIO) was used to extract total RNA from BMMs and C3H10 cells, as well as from the bones of mice used in the OVX experiment. The HiFiScipt cDNA Synthesis kit (CWBIO) was used to reverse transcribed total RNA into cDNA, and the cDNA was amplified via quantitative real-time PCR. qPCR was performed using the UltraSYBR Mixture (Takara Bio, Otsu, Japan) with the ABI Prism 7500 Fast System (Applied Biosystems, Foster City, CA, USA) under the following PCR cycling conditions: 95 °C for 5 min, 40 cycles at 95 °C for 10 s, 60 °C for 30 s, followed by a final step at 4 °C for 10 min. GAPDH was used as endogenous control, and the expression level of each gene was calculated using the 2 − ΔΔCt method. The primer sequences are listed in Table 1.

### Protein extraction and Western blotting

Total protein was extracted from BMMs and C3H10 cells and from the bones of mice used in the OVX experiment using a radioimmunoprecipitation assay lysis buffer (RIPA) (Sigma-Aldrich), and the protein concentration was measured using a bicinchoninic acid protein (BCA) assay kit (EMD Millipore, Bedford, MA, USA). Protein equivalents were separated using 10% SDS PAGE and transferred onto PVDF membranes (EMD Millipore). The membranes were blocked for an hour at room temperature with 5% skim milk in Tris-buffered saline with Tween 20. After incubating primary antibodies overnight at 4 °C, the membranes were nurtured with corresponding HRP conjugated secondary antibodies (Proteintech Group, Chicago, IL, USA.) The bands were visualized employing ECL Luminous Liquid (EMD Millipore). ImageJ software was used to compute the relative grey level of proteins (NIH). Anti-TRAIL was procured from Santa Cruz Bio (Santa Cruz, CA, USA). Anti-CTSK, TRAP, NFATc1, OPG, RUNX2, OCN, RANKL, COL1a, p-P65, P65, p-P38, P38, p-JNK, JNK, p-ERK, ERK, p-IKBα, IKBα, IKKα, IKKβ, and p-IKKα/β were all purchased from Cell Signaling Technology (Danvers, MA, USA).

### Fluorescent staining and actin ring formation of osteoclasts

The BMMs were cultivated in a 96-well plate and stimulated for osteoclastogenesis. The cells were then fixed in 4% PFA for 15 min before being broken into 0.1% Triton X-100 for 30 min. Anti-TRAIL antibody (1:200) was added to cells overnight at 4 °C after blocking in 10% goat serum for an hour. After rinsing with PBS, the fluorescent secondary antibody was added and incubated for an hour at room temperature (Alexa Fluor 488, green). The F-actin ring was stained with rhodamine phalloidin at room temperature for an hour in a dark room. The cells were then rinsed with PBS and stained with DAPI. Finally, the cells were observed using a fluorescence microscope (NIKON TE2000, Nikon Corporation, Minato, Tokyo, Japan).

### Co-immunoprecipitation (IP) assay

The Co-IP experiment was conducted according to the protocol of the Co-IP kit (Thermofisher Scientific, MA, USA). Briefly, the cell lysates were immunoprecipitated with 2 μg anti-TRAIL antibody at 4 °C overnight and then incubated for an hour at room temperature with 0.25 mg protein A/G-agarose. The magnetic beads were then eluted using Elution Buffer, magnetically separated, and the supernatant containing the anti-TRAIL antibody was saved. The bound proteins were resolved using 8% SDS-PAGE and then incubated with anti-TRAIL and anti-ubiquitin.

### The OVX-induced bone loss model

Eight-week-old C57BL/6 female mice (n = 15) weighing 20–25 g were anesthetized with 3 mg/mL pentobarbital for bilateral ovariectomy (OVX). The mice were randomly assigned to 3 groups of 5 mice each. The sham and vehicle groups received corn oil (MedChemExpress, NJ, USA) containing 10% DMSO (MedChemExpress), whereas the Tetrandrine group received tetrandrine (30 mg/kg) every 3 days for 42 days. The isolated femur and tibia were then fixed in 4% PFA for 48 h before micro-CT and histological experiments.

### Enzyme-linked immunosorbent assay

The serum of mice was collected, and the enzyme-linked immunosorbent assay of IL-6, TNFα, OPG, and RANKL in the serum was performed with the ELISA kit (ProteinTech Group) according to the manufacturer’s instruction.

### Micro-CT scanning

A previously published approach was employed to analyze the fixed tibia using a micro-CT scanner (1072 μCT System; Sky Scan, Bruker microCT, Kontich, Belgium) (Boerckel et al. 2014). The following parameters are set: X-ray energy and current were 70 kV and 300 μA, respectively, the aluminum filter was 0.5 mm, the rotation step was 0.4°, and the isometric resolution was 9 μm. The NRecon program (Bruker microCT, kontich, Belgium) was used for reconstruction. For rebuilding, the settings used were as follows: (1) smoothing; (2) ring artifact correction; (3) 40% beam hardening correction. Each sample was evaluated utilizing the software CTAn (Bruker microCT, Kontich, Belgium) for BV/TV (trabecular bone volume per total volume), Tb.N (the trabecular number), Tb.Th (the trabecular thickness), and Tb.Sp (trabecular separation).

### Histological analysis

The fixed femur and tibia were immersed in 10% ethylenediaminetetraacetic acid (EDTA), decalcified for 2 weeks at 37 °C, and then embedded in paraffin to prepare the histological sections. These sections were stained with Masson trichrome, TRAP, and hematoxylin–eosin (HE) staining solutions and then photographed using the Aperio Scanscope high-quality microscope (MT Waverley, Vic, Australia). Image Pro Plus software was used to calculate the number of trap-positive cells (Media Cybernetics, Bethesda, MD, USA).

### Calcein-alizarin red S labeling and von Kossa staining

On days 0 and 4, 20 mg/kg calcein (Sigma, C0875-5 g, 1 mg/mL in 2% NaHCO_3_ solution) and 40 mg/kg alizarin red S (Sigma, a5533-25 g, 2 mg/mL in H_2_O) were injected intraperitoneally. The mice were euthanized on the seventh day, and their bones were fixed, dehydrated, and embedded with embedded-812 (Electron Microscope Science). The samples were cut into 5-μm slices with a hard tissue cutter (RM2265, Leica, Wetzlar, Germany). Fluorescent labeling images were obtained with a microscope (BX51, Olympus).

For von Kossa staining, the femur samples were sliced into 6-μm dense pieces. Subsequently, the sections were irradiated with ultraviolet light in 2% silver nitrate solution for 1 h before being incubated with 5% sodium thiosulfate for 2 min. Finally, using a Diplan light microscope, the staining results were examined (Leitz).

### Statistical analysis

The data from triplicate trials were evaluated utilizing Graphpad Prism 9 windows (Graphpad Software Inc., San Diego, CA, USA) and expressed as the mean ± SEM. For group comparison, one-way or two-way ANOVA and the Student’s t-test were used, with P < 0.05 indicating a statistically significant difference.

## Results

### Tetrandrine inhibits RANKL-induced osteoclasts formation and bone resorption of BMMs

**Fig. 1:**
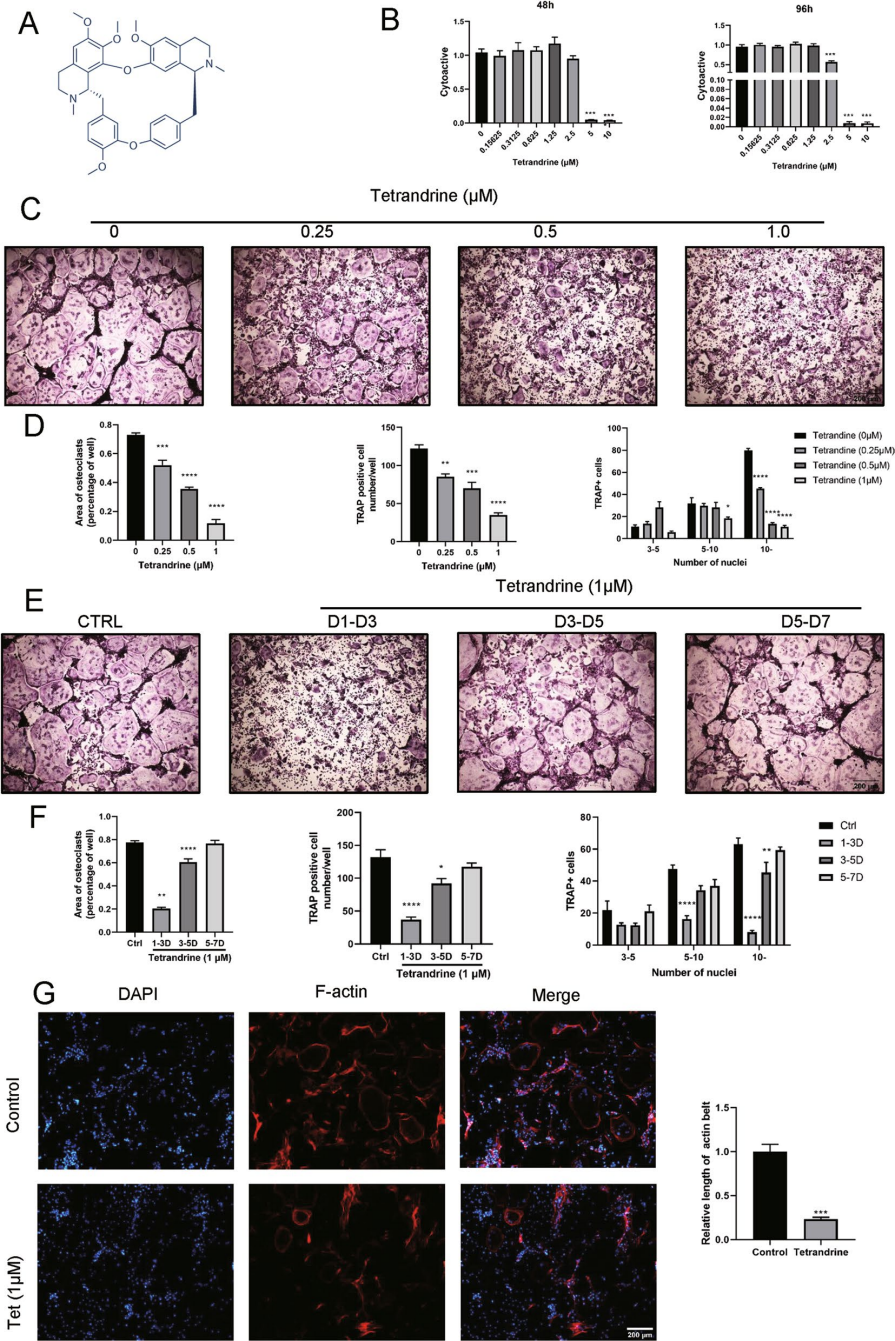
Tetrandrine inhibit RANKL-induced osteoclastogenesis. **A** The chemical structural formula of Tetrandrine; **B** Tetrandrine impact on BMMs viability was detected by CCK-8 assay at 48 h and 96 h. **C** With different dilutions of Tetrandrine, M-CSF and RANKL for 7 days, BMMs were processed; scale bar = 200 μm. **D** The number and area of TRAP-positive cells. **E** TRAP-positive BMMs after the treatment with 1 μM Tetrandrine for the designated days in the process of osteoclastogenesis; scale bar = 200 μm. **F** To quantify TRAP-positive cells, the number and area of osteoclasts. **G** Immunofluorescence image for showing the formation of F-actin. All experiments were carried out in triplicate. The results were presented as mean ± SEM. *P < 0.05, **P < 0.01, ***P < 0.005 and ****P < 0.001 as compared with the control group

**Fig. 2:**
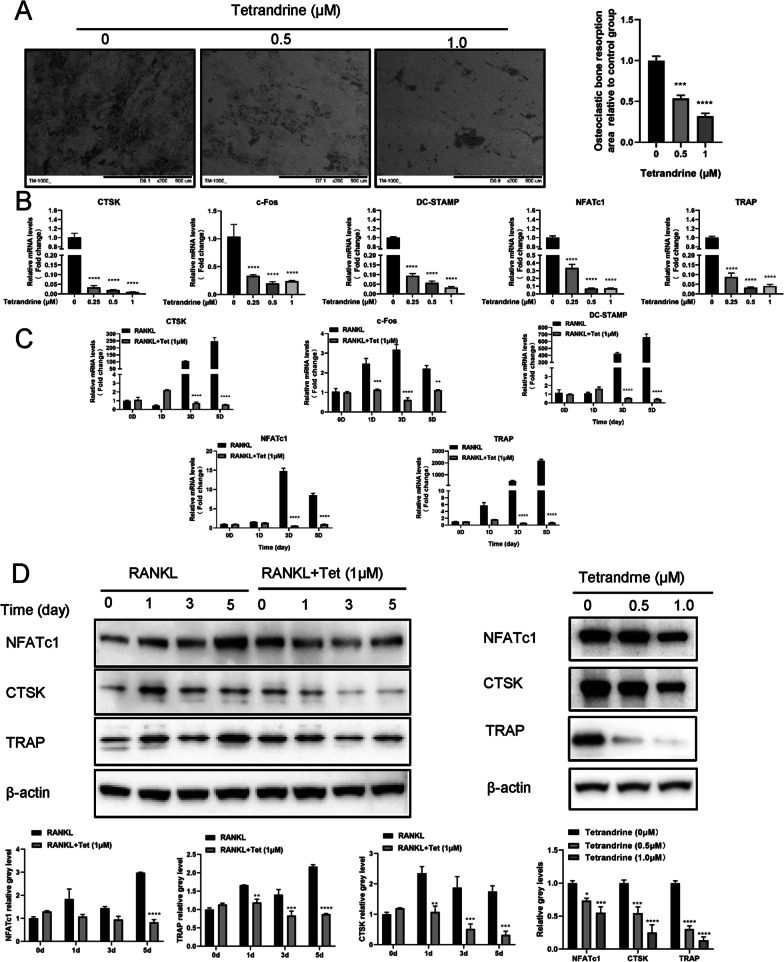
Tetrandrine inhibits osteoclastic bone resportion and expression of osteoclastogenesis related genes. **A** BMMs were planted into slices of bone and treated with different concentrations of Tetrandrine for 7 days and bone resorption pit pictures taken by a scanning electron microscope are presented; scale bar = 500 μm. The pit zone resorption area were shown by the ImageJ. **B** The detection of expression levels of osteoclastogenesis related genes in BMMs with 0, 0.25, 0.5, 1 μM was carried out with qPCR. **C** After treating BMMs with Tetrandrine (1 μM) for 0, 1, 3, 5 days, they were detected via RT-qPCR. The mRNA expression levels of NFATc1, c-Fos, DC-STAMP, TRAP, CTSK were evaluated.** D** For 0, 1, 3, 5 days with or without 1 μM of Tetrandrine, the treatment of BMMs was carried out with RANKL. The protein expression levels of NFATc1, TRAP and CTSK were evaluated. All experiments were carried out in triplicate. The results were presented as mean ± SEM. *P < 0.05, **P < 0.01, ***P < 0.005 and ****P < 0.001 as compared with the control group

The chemical structure formula of Tetrandrine is shown in Fig. 1A. The CCK8 experiment was performed to observe the drug’s toxicity on BMMs. The results showed that when the Tetrandrine concentration exceeded 2.5 μM, the proliferative activity of BMMs was significantly inhibited (Fig. 1B). Trap staining showed that Tetrandrine significantly inhibited the osteoclast formation of BMMs in a dose-dependent manner (Fig. 1C). The area and number of osteoclasts decreased as the Tetrandrine concentration increased (Fig. 1D). Tetrandrine also decreased the number of trap-positive cells with 5 or more nuclei, confirming its inhibitory effect on osteoclast formation (Fig. 1D). The trap staining findings likewise revealed that Tetrandrine had the most noticeable effect on the early stage (days 1–3) of BMMs osteoclastogenesis, some effects on the middle stage (days 3–5), but had almost no effect on the late stage (days 5–7) (Fig. 1D, E). In addition, we analyzed the formation of the F-actin ring by immunofluorescence, which is critical for the function of osteoclasts, and discovered that Tetrandrine significantly reduced the formation of the F-actin ring compared to the control group (Fig. 1G). Furthermore, the results of the bone resorption experiment indicated that the absorptive capacity of osteoclasts decreased as the Tetrandrine concentration increased (Fig. 2A). These findings indicated that Tetrandrine impedes RANKL-induced osteoclastogenesis of BMMs.

### Tetrandrine inhibits the expression of osteoclastogenesis-related genes

We evaluated the expression of osteoclast differentiation-associated genes to understand the fundamental molecular mechanism of the anti-osteoclastogenic effect of Tetrandrine on BMMs. Tetrandrine inhibited the mRNA expression of CTSK, c-Fos, DC-STAMP, NFATc1, and TRAP in a dose-dependent manner (Fig. 2B). Moreover, the expression of these osteoclastogenesis-related genes increased under the stimulation of RANKL alone but decreased significantly after increasing Tetrandrine stimulation in the same period (Fig. 2C). Tetrandrine was also found to downregulate the protein expression levels of NFATc1, CTSK, and TRAP using Western blotting (Fig. 2D). Tetrandrine prevalently inhibited the expression of osteoclastogenesis-related genes.

### Tetrandrine affects osteoclastogenesis by promoting the ubiquitination of TRAIL

**Fig. 3:**
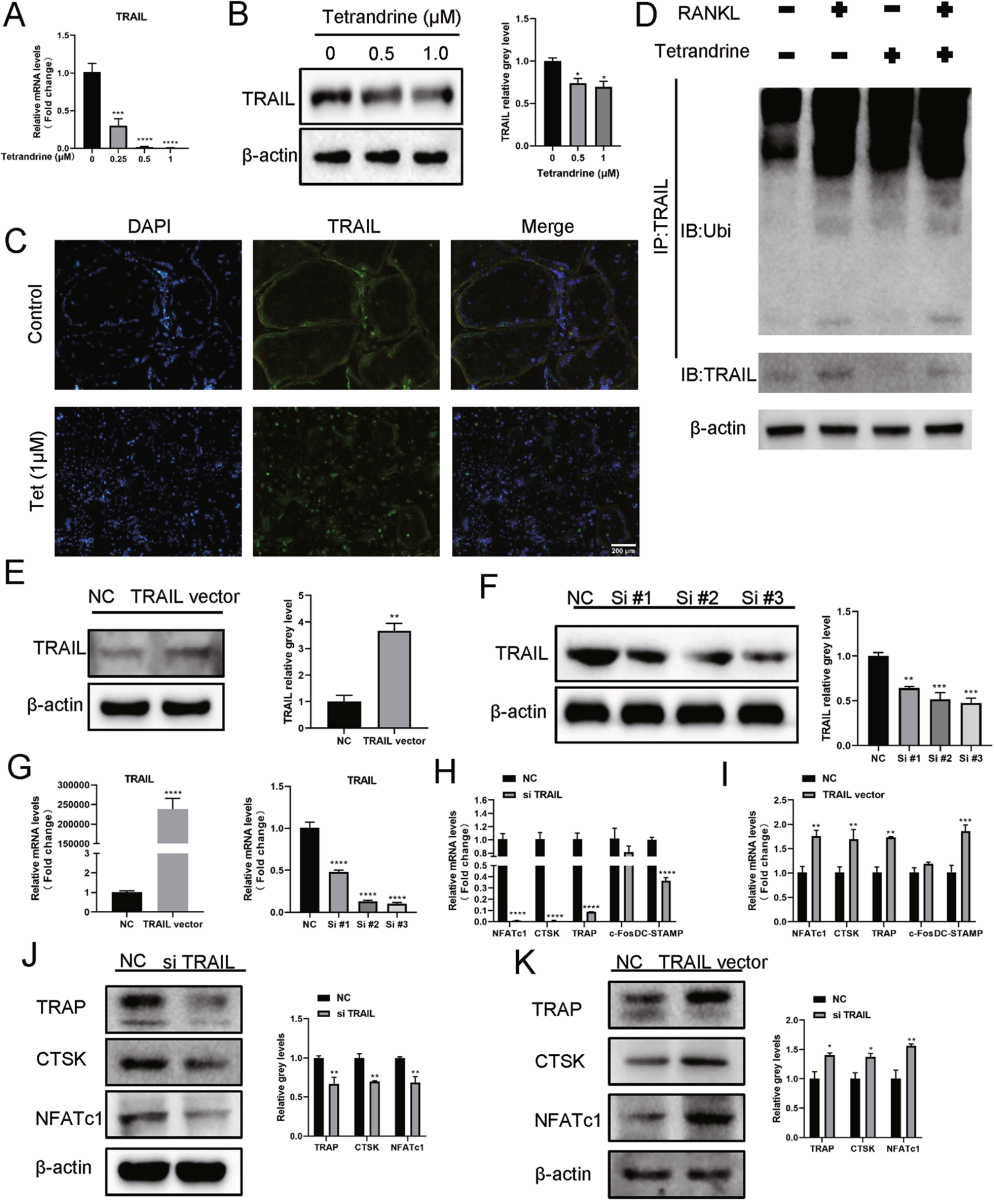
Tetrandrine affects osteoclastogenesis by promoting the ubiquitination of TRAIL. **A** The mRNA expression level of TRAIL in BMMs with 0, 0.25, 0.5, 1 μM of Tetrandrine was carried out with RT-qPCR. **B** The expression of level of TRAIL in BMMs treated with specified concentrations of Tetrandrine for 5 days was detected using western blot. **C** Immunofluorescence image for showing TRAIL expression in cytoplasm; scale bar = 200 μm. **D** The co-IP assay was used to detect the effect of Tetrandrine on TRAIL ubiquitination with or without RANKL. **E–G** The transfection efficiency of TRAIL silencing (si #1, si #2, si #3) and TRAIL overexpression (TRAIL vector) detected via RT-qPCR and western blotting. **H–K** BMMs were transfected with siTRAIL and TRAIL vector, the expression of the related genes and proteins that were revealed by RT-qPCR and western blotting. All experiments were carried out in triplicate. The results were presented as mean ± SEM. *P < 0.05, **P < 0.01, ***P < 0.005 and ****P < 0.001 as compared with the control group

**Fig. 4:**
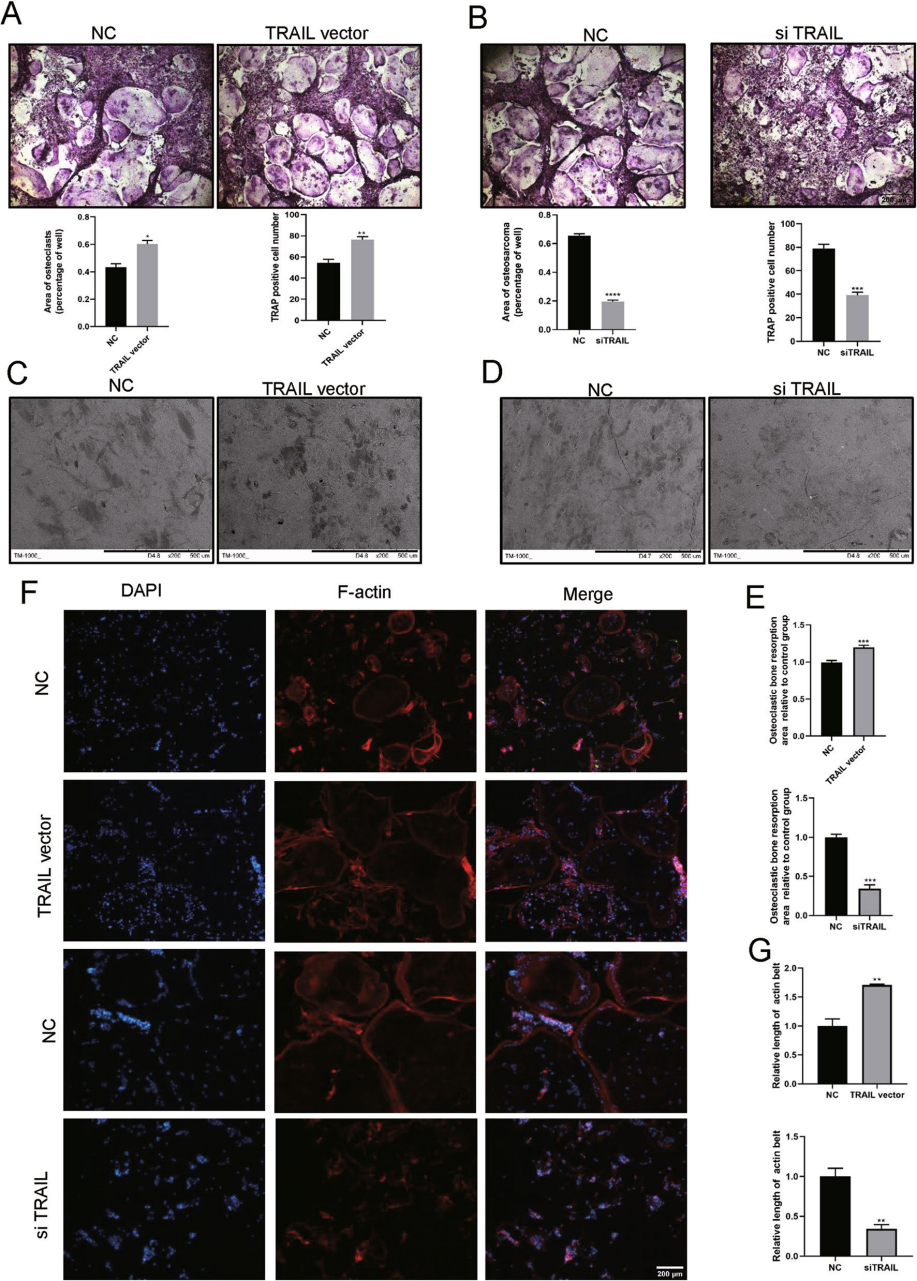
TRAIL overexpression or silencing can promote and inhibit osteoclast differentiation and bone resorption, respectively. **A**, **B** The number and area of TRAP-positive BMMs following transfection with TRAIL vector or siTRAIL; scale bar = 200 μm. **C**–**E** The bone resorption pits of BMMs following transfection with siTRAIL or TRAIL vector observed with SEM; scale bar = 500 μm. The pit zone resorption area were shown by the ImageJ. **F**, **G** The effect of siTRAIL and TRAIL vector on the F-actin ring formation; scale bar = 200 μm. All experiments were carried out in triplicate. The results were presented as mean ± SEM. *P < 0.05, **P < 0.01, ***P < 0.005 and ****P < 0.001 as compared with the control group

Previous research has shown that TRAIL is important in osteoclastogenesis (Sambandam et al. 2016; Yen et al. 2012) and Tetrandrine inhibited TRAIL expression in BMMs (Fig. 3A–C). Furthermore, TRAIL ubiquitination was accelerated by Tetrandrine administration, which was thereafter degraded (Fig. 3D). We transfected BMMs with TRAIL siRNA and TRAIL vector to study the effect of TRAIL on RANKL-induced osteoclastogenesis. TRAIL silencing and overexpression were verified by Western blotting and RT-qPCR. TRAIL expression increased considerably following TRAIL vector transfection when compared to the NC group (Fig. 3E, G). Similarly, TRAIL siRNA transfection resulted in successful TRAIL silencing, especially in terms of the effect of Si-3# (Fig. 3F, G). As a result, in the next investigations, we employed siTRAIL-3# to inhibit TRAIL expression. TRAIL silencing downregulated osteoclastogenesis-related gene expression and vice versa (Fig. 3H–K). TRAP staining demonstrated that, as compared to the NC group, TRAIL overexpression increased the area of osteoclasts and TRAP-positive cell number (Fig. 4A), but TRAIL silencing decreased the area of osteoclasts and TRAP-positive cells number (Fig. 4B). In addition, TRAIL overexpression and silencing increased and decreased osteoclast bone resorption capacity (Fig. 4C–E). TRAIL overexpression considerably supported the formation of the F-actin ring compared to that in the NC group, whereas TRAIL silencing had the opposite effect (Fig. 4F, G). These results proposed that Tetrandrine’s inhibitory effect on osteoclastogenesis may be achieved by affecting the TRAIL expression.

### TRAIL overexpression partially rescued the inhibitory effect of Tetrandrine on osteoclastogenesis

**Fig. 5:**
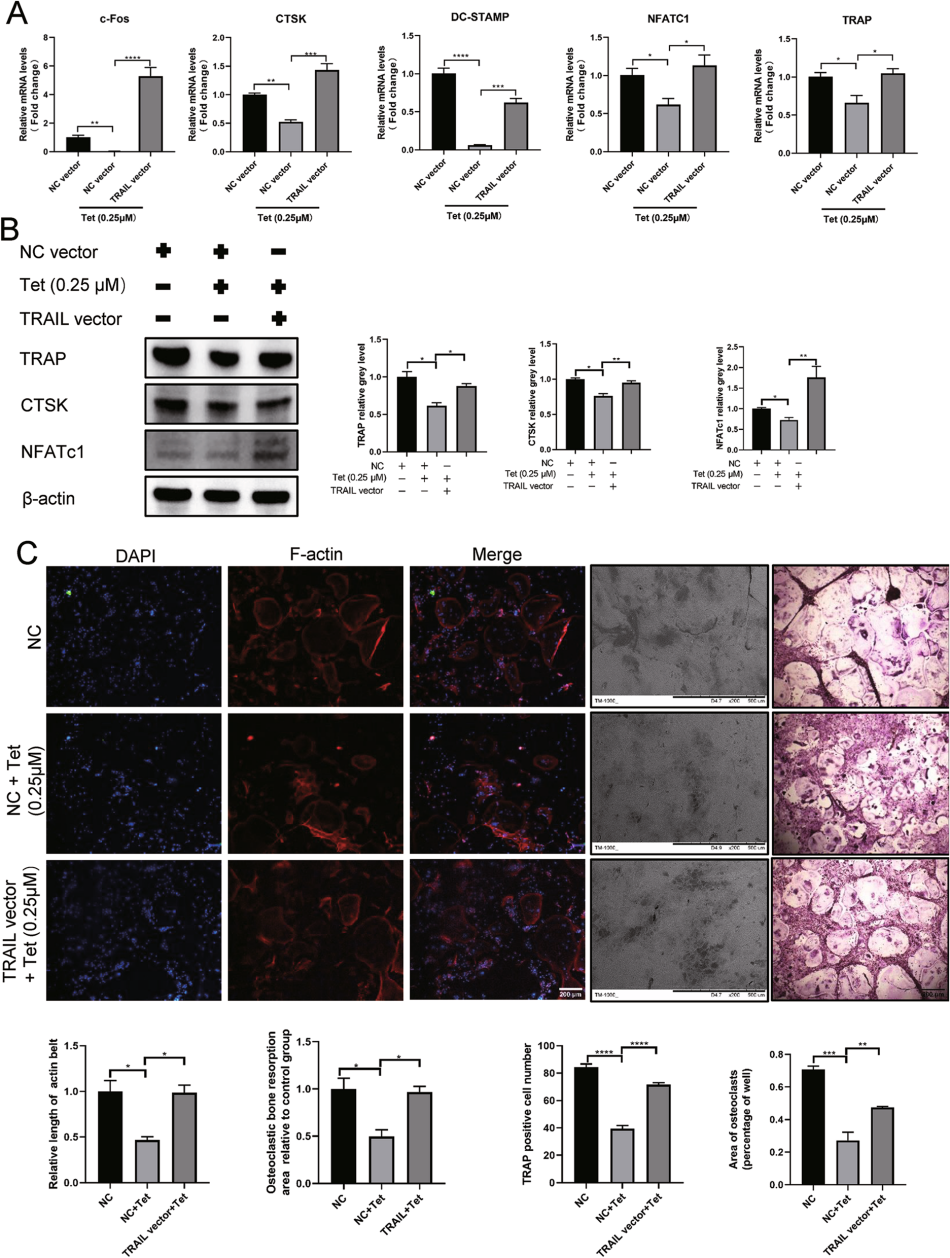
TRAIL overexpression partially rescued the inhibitory effect of Tetrandrine on osteoclastogenesis. **A** The mRNA expression of c-Fos, CTSK, DC-STAMP, NFATc1 and TRAP in BMMs in rescue experiment was detected by RT-qPCR. **B** Western blotting demonstrated the expression of NFATc1, TRAP and CTSK in BMMs in rescue experiment. **C** The number and area of TRAP-positive cells, the bone resorption pits and F-actin formation in rescue experiment; Scale bar in TRAP staining and immunofluorescence image = 200 μm; scale bar in bone resorption pit picture = 500 μm. All experiments were carried out in triplicate. The results were presented as mean ± SEM. *P < 0.05, **P < 0.01, ***P < 0.005 and ****P < 0.001 as compared with the control group

As shown in Fig. 5A and B, qPCR and Western blotting confirmed that TRAIL overexpression attenuated the downregulation of osteoclastogenesis-related genes caused by Tetrandrine. Furthermore, using immunofluorescence (first three lines), bone resorption (fourth line), and TRAP staining (fifth line), we found that Tetrandrine inhibited the formation of F-actin, osteoclastogenesis, and osteoclast bone resorption when compared to that in the NC group. Intriguingly, the inhibitory effect was diminished following concurrent TRAIL overexpression (Fig. 5C).

### Tetrandrine inhibits NF-κB and MAPK pathways by downregulating TRAIL expression

**Fig. 6:**
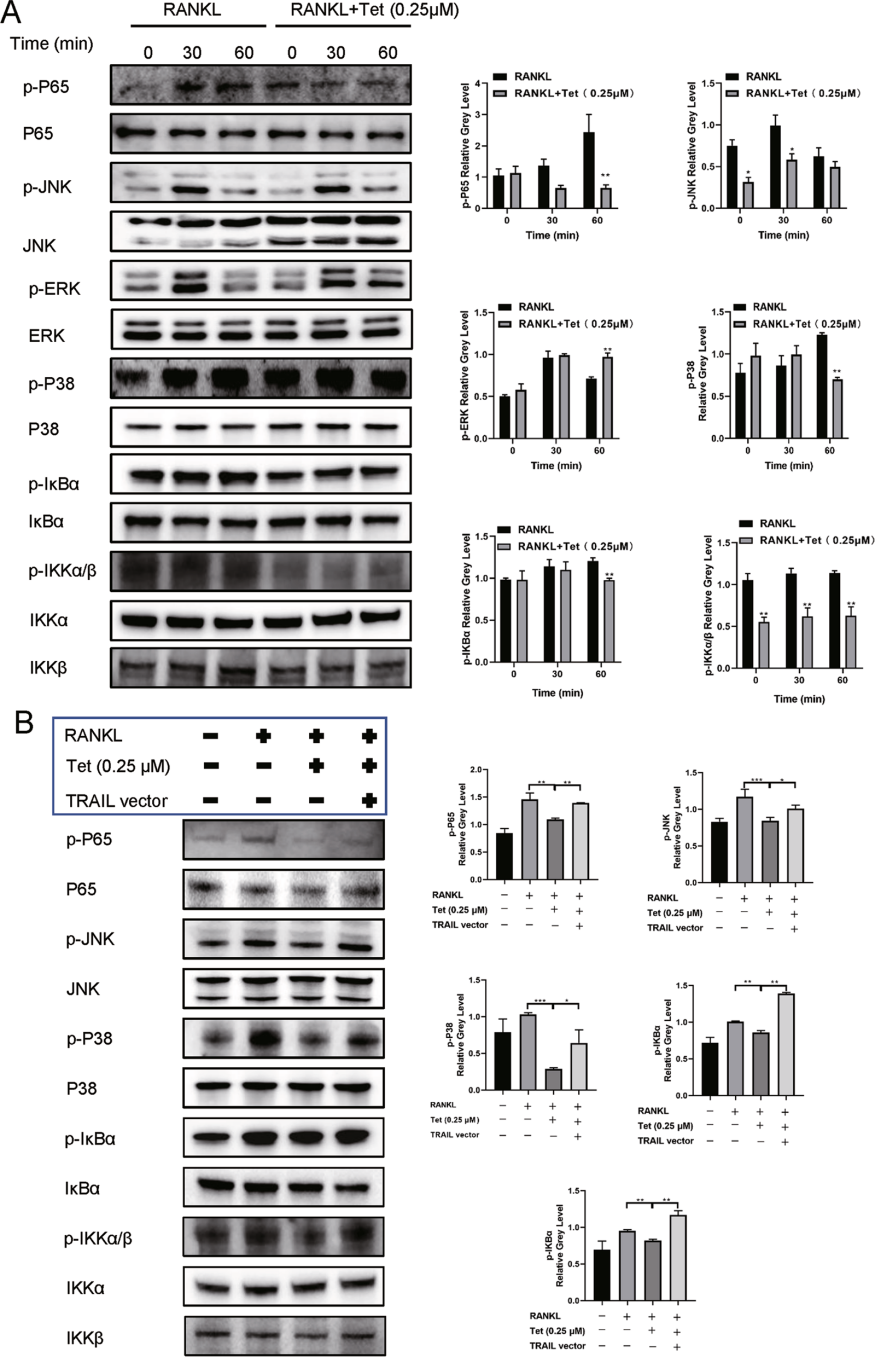
Tetrandrine inhibits NF-κB and MAPK pathways by downregulating TRAIL expression. **A** Treatment of BMMs was carried out with RANKL, with or without 0.25 μM Tetrandrine for 0, 30 and 60 min. The p-P65, p-JNK, p-ERK, p-P38, p-IκBα, p-IKKα/β protein levels were evaluated. **B** The p-P65, p-JNK, p-P38, p-IκBα, p-IKKα/β protein levels were determined by western blotting in rescue experiment. All experiments were carried out in triplicate. The results were presented as mean ± SEM. *P < 0.05, **P < 0.01, ***P < 0.005 and ****P < 0.001 as compared with the control group

The NF-κB and MAPK pathways are both significant in RANKL-induced osteoclastogenesis (Yang et al. 2021; Zeng et al. 2013). To investigate how Tetrandrine influences osteoclast differentiation via TRAIL, we assessed the expression levels of related proteins to explore whether the NF-κB and MAPK pathways are involved in the molecular mechanism. BMMs were treated with RANKL for 0, 30, and 60 min, with or without Tetrandrine. When Tetrandrine was added to the RANKL-stimulated group, the phosphorylation of p38, p65, JNK, IKBα, and IKKα/β decreased significantly (Fig. 6A). Interestingly, TRAIL upregulation weakened Tetrandrine’s inhibitory effect on the NF-κB and MAPK pathways (Fig. 6B). These data illustrated that TRAIL was critical in the Tetrandrine production process.

### Tetrandrine promotes osteoblastogenesis of C3H10

**Fig. 7:**
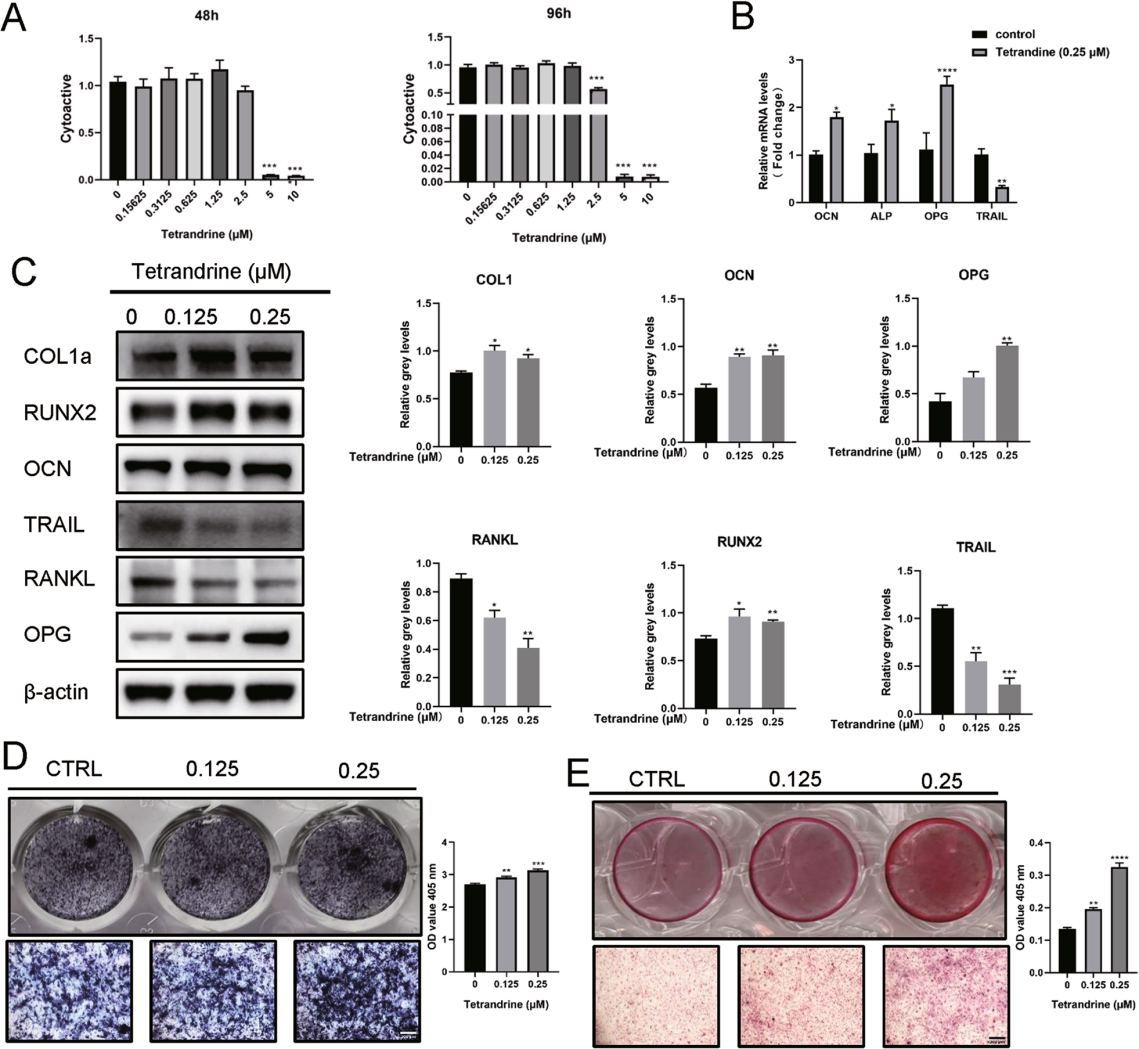
Tetrandrine promotes C3H10 osteoblastogenesis and inhibits TRAIL expression in C3H10. **A** Effect of Tetrandrine on C3H10 viability by CCK-8 assay at 48 h and 96 h. **B** The TRAIL, OCN, ALP and OPG expression levels in C3H10 treated with Tetrandrine were detected with RT-qPCR. **C** The protein levels of COL1a, RUNX2, OCN, TRAIL, RANKL and OPG were measured with western blotting. **D**, **E** ALP and ARS expression in C3H10 following the treatment with various concentration of Tetrandrine for 7 days. The mineralized extracellular matrix was detected with Alizarin Red S staining for 14 days. The OD values obtained for ALP solutions and mineralized matrix solutions after their treatment with Tetrandrine; scale bar = 200 μm. All experiments were carried out in triplicate. The results were presented as mean ± SEM. *P < 0.05, **P < 0.01, ***P < 0.005 and ****P < 0.001 as compared with the control group

Tetrandrine’s effects on osteoblast differentiation were evaluated. Tetrandrine (0–10 μM) was applied to C3H10 cells for 48 and 96 h, and its cytoactivity was determined using the CCK-8 assay (Fig. 7A). Tetrandrine depressed the TRAIL expression in C3H10 cells while increasing the expression of osteoblast-related genes increased, including OCN, ALP OPG, COL1a, and RUNX2 (Fig. 7B, C). RANKL is necessary for the expression of typical genes in osteoclasts and is mainly secreted by osteoblasts (Boyle et al. 2003). Conversely, osteoblasts produce OPG, which acts as a decoy receptor to block RANKL–RANK interaction (Yahiro et al. 2020). Unexpectedly, after Tetrandrine treatment, the RANKL expression in C3H10 decreased, but the OPG expression increased, which was consistent with its experimental results in inhibiting osteoclast differentiation (Fig. 7C). Moreover, ALP and ARS staining of C3H10 cells revealed that Tetrandrine promotes osteoblast differentiation and mineralization (Fig. 7D, E). Tetrandrine can enhance C3H10 osteoblastogenesis while inhibiting the TRAIL expression.

### Tetrandrine prevents OVX-induced bone loss in vivo

**Fig. 8:**
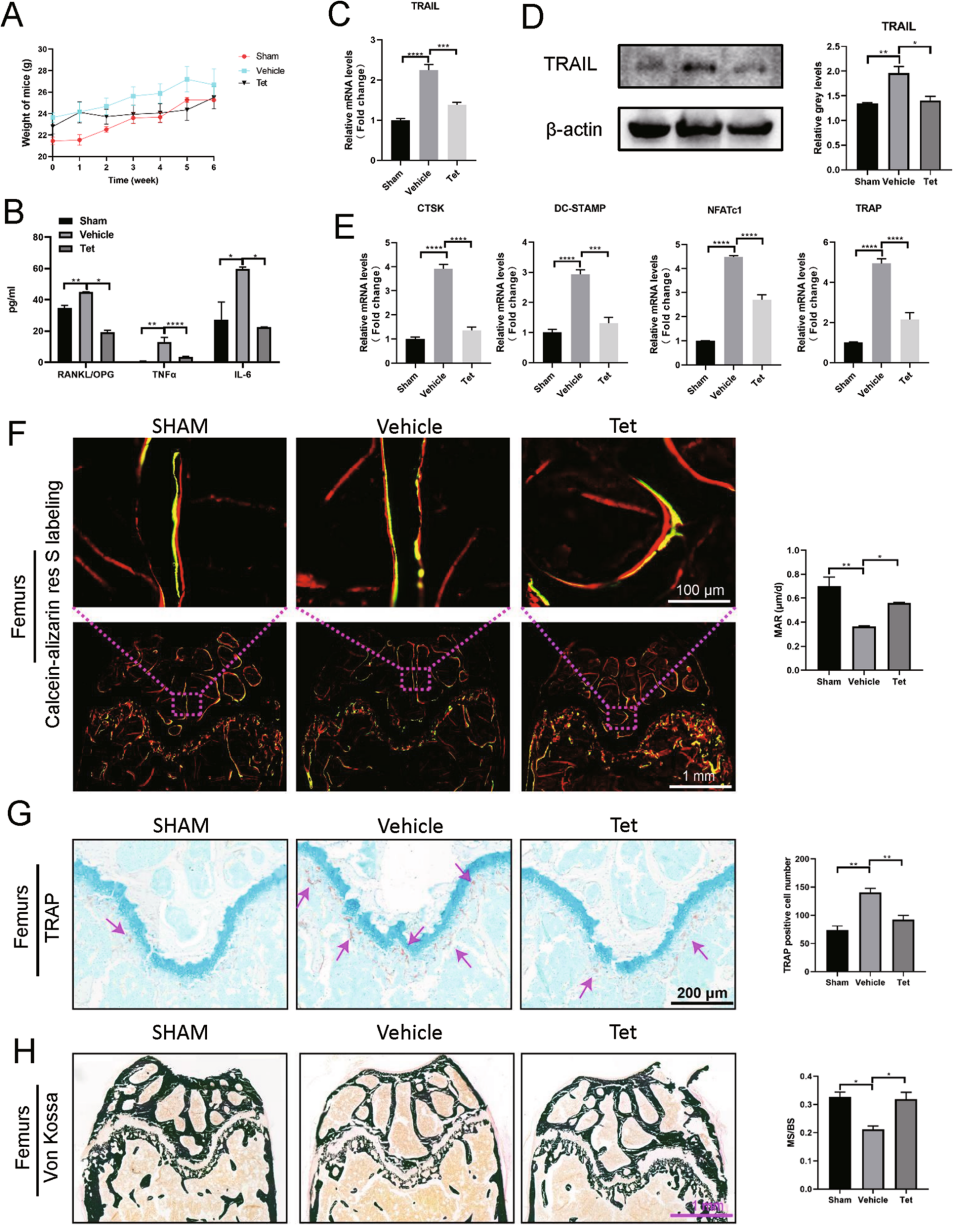
Tetrandrine prevents OVX-induced bone loss in vivo. **A** Weight change diagram of mice after Tetrandrine treatment (each; n = 5). **B** RANKL/OPG, TNFα and IL-6 levels in serum of mice were determined with Elisa assay. **C**, **D** Detection of the tetrandrine effects on the expression of TRAIL in the OVX-induced model carried out with RT-qPCR and western blot. **E** Detection of NFATc1, DC-STAMP, TRAP and CTSK expression levels was carried out with RT-qPCR. **F** Calcein-alizarin red S labeling to detect the femur mineral apposition rate (MAR) in mice. **G** TRAP staining was used for sections of femurs and number of osteoclasts per field of each specimen were measured. **H** The Von kossa staining was used for the sections of femurs and the MS/BS (%) was measured. All experiments were carried out in triplicate. The results were presented as mean ± SEM. *P < 0.05, **P < 0.01, ***P < 0.005 and ****P < 0.001 as compared with the control group

**Fig. 9:**
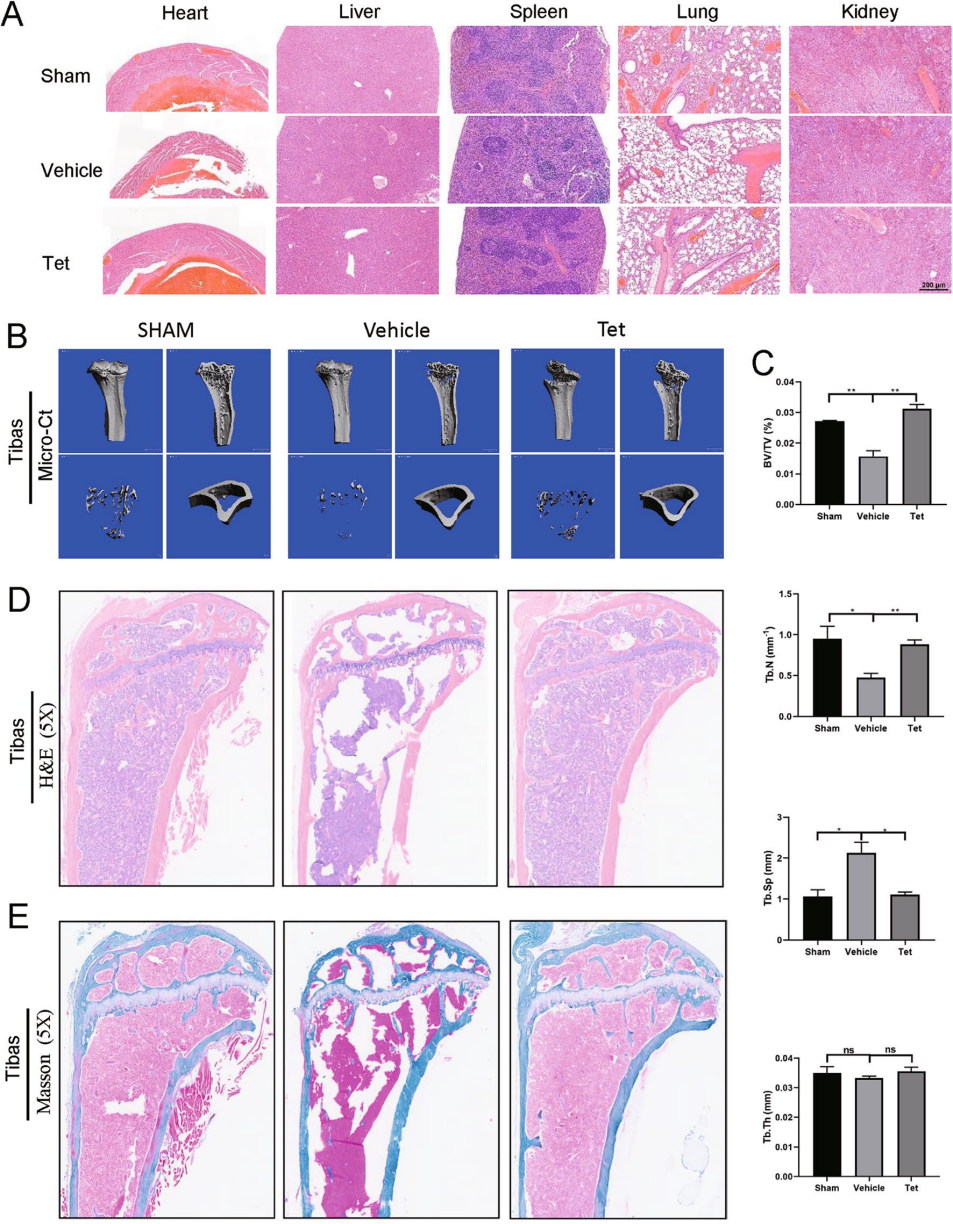
Tetrandrine inhibits OVX-induced osteoporosis in mice model. **A** H&E staining was used for several major organs of mice in three groups (each; n = 5). **B**, **C** The micro-CT was used for the scanning tibias of each mouse, and the BS/BV, Tb.N, Tb.Sp and Tb.Th values were recorded for each specimen. **D**, **E** H&E and Masson staining was also used for the femurs. All experiments were carried out in triplicate. The results were presented as mean ± SEM. *P < 0.05 and **P < 0.01 as compared with the control group

Based on the findings, we performed bilateral OVX in mice as an in vivo experimental model of postmenopausal osteoporosis. The weight of mice in the Tetrandrine-treated group was not inhibited by the administration, and no toxicity was identified in the major organs (Figs. 8A, 9A). TNFα and IL-6 have been shown to promote osteoclast differentiation while inhibiting osteoblast formation (Li et al. 2020). Tetrandrine inhibited RANKL/OPG, TNFα, and IL-6 upregulation in the serum of OVX group mice (Fig. 8B). RNA and protein were extracted from the isolated mice forelimbs. As expected, the TRAIL expression level in the OVX group significantly increased when compared to that in the sham group. After Tetrandrine treatment, the TRAIL expression was inhibited (Fig. 8C, D). The results confirmed that TRAIL downregulation played an important role in the inhibition of bone loss by Tetrandrine. Moreover, the level changes of osteoclastogenesis-related genes corresponded to the results of in vitro experiments (Fig. 8E).

The micro-CT study showed significant bone loss in the tibias of OVX-induced osteoporosis animals. Although the value of Tb.Th was not statistically different, the bone quality indicators such as the values of BV/TV and Tb.N were increased by Tetrandrine, but the Tb.Sp value was reduced (Fig. 9B). Moreover, H&E and Masson staining revealed considerable bone loss in the OVX model group as compared to that in the control group. However, Tetrandrine treatment increased the thickness and number of the trabecular bone (Fig. 9D, E). Similarly, TRAP staining revealed that OVX mice had more TRAP-positive mature osteoclasts, but Tetrandrine-treated mice had fewer stained osteoclasts (Fig. 8G). Von Kossa staining and calcein–alizarin red S labeling were used to examine the femoral mineralization area and deposition rate, respectively to further investigate the influence of Tetrandrine on osteoblast development. Tetrandrine significantly enhanced the mineral apposition rate (MAR) of the femur as compared to the vehicle group, according to calcein-alizarin red S labeling (Fig. 8F). The von Kossa staining of femurs revealed that the ratio of the mineralized area (black part of the trabecular bone region) to the bone area of the distal femur decreased in the OVX group, but Tetrandrine treatment improved this ratio (Fig. 8H). Taken together, our findings demonstrated that Tetrandrine could inhibit osteoclast activity while promoting osteoblast activity, thereby reducing OVX-induced bone loss.

## Discussion

Although the synergistic effect of osteoclastic bone resorption and bone formation maintains bone integrity and mineral homeostasis, osteoclasts are responsible for several skeleton diseases, such as osteoarthritis, osteoporosis, and cancer-induced osteolysis (Boyce 2013; Jiang et al. 2021). Antiresorptive drugs including estrogen replacement, bisphosphonates, and denosumab have been widely adopted in clinical treatment to prevent bone loss (Miyazaki et al. 2014). However, atypical femoral fractures and osteonecrosis of jaw bones have been linked to osteoporosis therapy with bisphosphonates and denosumab (Boyce 2013; Palmerini et al. 2017). Therefore, additional treatments are required to alleviate various adverse effects of the existing treatments. Tetrandrine contains several biological activities, such as anti-inflammatory and anti-tumor effects (Gao et al. 2016; Xu et al. 2021). More research is required to explore the relationship between Tetrandrine and osteoporosis and its specific underlying mechanism.

In vivo and in vitro experiments confirmed the inhibitory effect of Tetrandrine on osteoclastogenesis. In addition, Tetrandrine inhibited TRAIL expression. Past articles have confirmed that decreased TRAIL can inhibit the differentiation of osteoclasts by inhibiting TRAF6 recruitment and ultimately inhibiting the activation of the NF-κB pathway (Gao et al. 2021). Moreover, we confirmed that the upregulation and downregulation of TRAIL expression in BMMs promoted and inhibited osteoclastogenesis, respectively. Therefore, we speculated that Tetrandrine inhibited osteoclastogenesis by reducing TRAIL expression. We also found that tetrandrine treatment accelerated ubiquitination of TRAIL, which was then degraded (Fig. 3D). It is both motivating and invigorating because TRAIL upregulation partially rescued osteoclastogenesis suppression caused by Tetrandrine treatment. The RANK/RANKL signaling pathway is a classical pathway for osteoclastogenesis, with the crucial first step being that RANKL recruits TRAF6 by interacting with its receptor (Boyle et al. 2003; Yamashita et al. 2007). Various studies have shown that RANKL-induced osteoclastogenesis is related to the activation of the NF-κB and MAPK (mainly composed of p38, ERK, and JNK) signaling pathways (Li et al. 2011; Wei et al. 2013). The NF-κB protein normally resides in the cytoplasm, where it interacts with inhibitory factors κB form complex (IκB) (An et al. 2016), and the IκB kinase (IKK) complex is involved in transcriptional activation by phosphorylating IκBα, thereby activating NF-κB (Chariot 2009). Our findings showed that Tetrandrine dampened the phosphorylation of IKKα, IKKβ, IκBα, P65, P38, and JNK (Fig. 6A), suggesting that Tetrandrine inhibited osteoclastogenesis via repressing the NF-κB and MAPK pathways. Moreover, we observed that TRAIL upregulation attenuated the inhibitory effect of Tetrandrine on both pathways (Fig. 6B). This result further confirmed that Tetrandrine affected osteoclastogenesis via TRAIL expression.

In addition to the inhibitory effect of Tetrandrine on osteoclastogenesis, it could promote osteoblast differentiation. Tetrandrine inhibited the TRAIL and RANKL expression in C3H10 while increasing the OPG expression. The decline in the ratio of RANKL to OPG also confirmed that Tetrandrine weakens osteoclastogenesis. Then, in the OVX-induced osteoporosis model, Tetrandrine had a significant therapeutic effect on bone loss and promoted bone formation, which is consistent with in vitro results.

## Conclusions

**Fig. 10:**
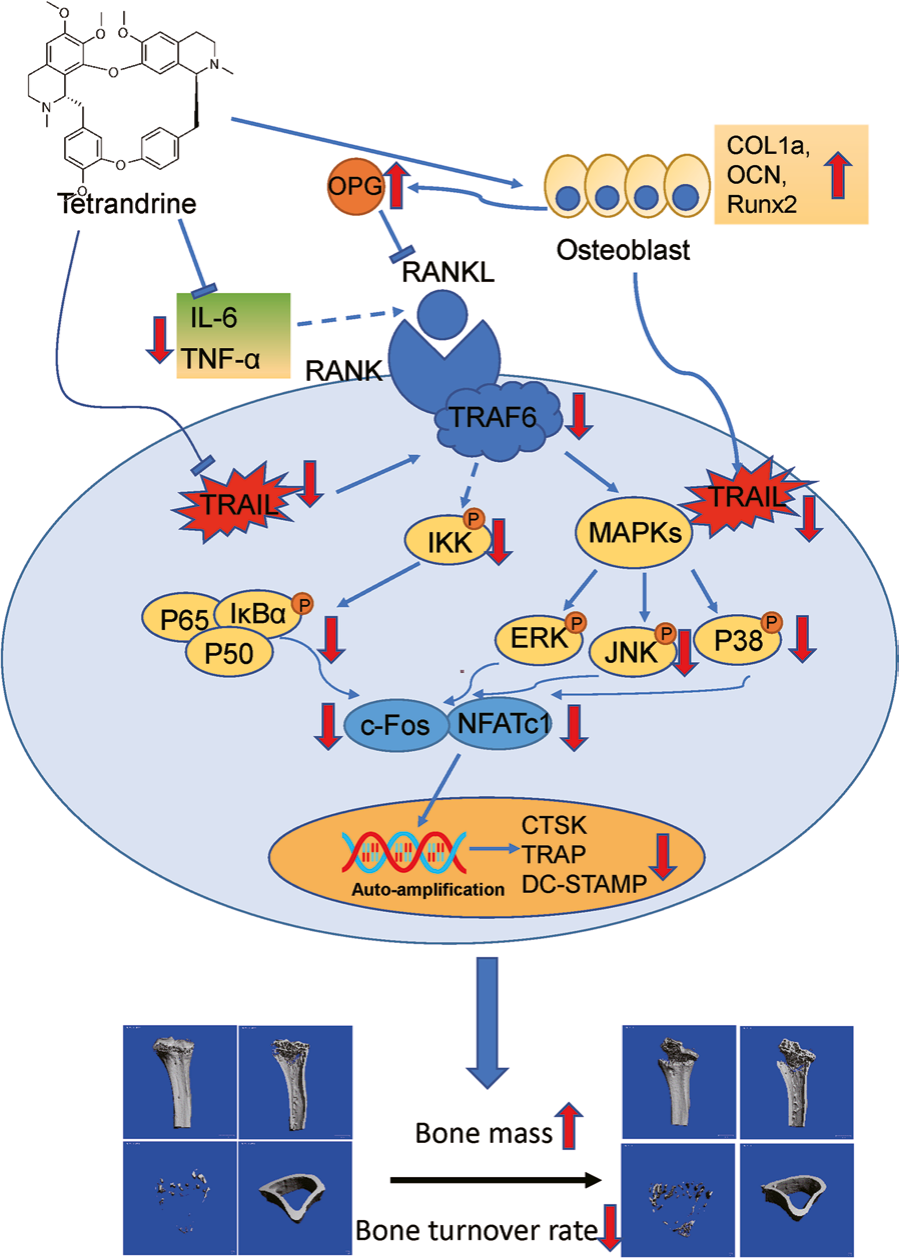
Schematic illustration for the mechanism by which Tetrandrine inhibits RANKL-induced osteoclastogenesis by promoting the degradation of TRAIL. RANKL recruits TRAF6 by interacting with its receptor RANK, and then activates downstream effectors such as NF-κB and MAPKs to promote mature osteoclast formation. Inflammatory factors including IL-6 and TNFα play an active role in the binding of RANK and RANKL and Tetrandrine has obvious anti-inflammatory effect. More importantly, Tetrandrine promotes TRAIL degradation and TRAIL downregulation inhibits the recruitment of TRAF6 by RANKL and eventually dampen osteoclast different and function. Besides, Tetrandrine can stimulate osteoblast difference

Overall, as shown in Fig. 10, we revealed, for the first time, that Tetrandrine could inhibit the activation of NF-κB and MAPK pathways by promoting TRAIL degradation, and finally inhibiting osteoclastogenesis. Furthermore, the drug significantly promoted osteogenesis. Therefore, Tetrandrine may be a potential antiosteopenia pharmacotherapy by efficiently preventing bone loss through the stimulation of bone formation and the inhibition of bone resorption so as to regulate the stability of bone remodeling.

## Data Availability

All data and materials generated or analyzed during this study are included in this article.

## Abbreviations

MCSF: Macrophage colony-stimulating factor
RANKL: Receptor activator of nuclear factor-κB ligand,
TRAIL: TNF-related apoptosis-inducing ligand
OPG: Osteoprotegerin
TRAF6: TNF receptor related factor 6
NF-κB: Nuclear factor-kB
NFATc1: Nuclear factor of activated T cells 1
TRAP: Tartrate resistant acid–base phosphatase
CTSK: Cathepsin K
DC-STAMP: Dendritic cell-specific transmembrane protein
TNFα: Tumor necrosis factor
BMMs: Bone marrow macrophages
ALP: Alkaline phosphatase
ARS: Alizarin red S
qPCR: Quantitative real-time polymerase chain reaction
RIPA: Radioimmunoprecipitation assay lysis buffer
BCA: Bicinchoninic acid
PFA: Paraformaldehyde
IP: Immunoprecipitation
BV/TV: Trabecular bone volume per total volume
Tb.N: Trabecular number
Tb.Th: Trabecular thickness
Tb.Sp: Trabecular separation
EDTA: Ethylenediaminetetraacetic acid
HE: Hematoxylin–eosin
OcS/BS: Osteoclast surface or bone surface area
IHC: Immunohistochemistry
EDTA: Ethylenediaminetetraacetic acid
OVX: Ovariectomy
